# Propagation Characteristics of Circular Airy Vortex Beams in a Uniaxial Crystal along the Optical Axis

**DOI:** 10.3390/mi13071006

**Published:** 2022-06-26

**Authors:** Guoliang Zheng, Qingyang Wu, Tiefeng He, Xuhui Zhang

**Affiliations:** 1 Sino-German College of Intelligent Manufacturing, Shenzhen Technology University, Shenzhen 518118, China; hetiefeng@sztu.edu.cn; 2 College of Big Data and Internet, Shenzhen Technology University, Shenzhen 518118, China; wuqingyang@sztu.edu.cn

**Keywords:** optical vortex, abruptly autofocusing beams, uniaxial crystal, spin-orbital coupling

## Abstract

Circular airy vortex beams (CAVBs) have attracted much attention due to their “abruptly autofocusing” effect, phase singularity, and their potential applications in optical micromanipulation, communication, etc. In this paper, we numerically investigated the propagation properties of circular airy beams (CABs) imposed with different optical vortices (OVs) along the optical axis of a uniaxial crystal for the first time. Like other common beams, a left-hand circular polarized (LHCP) CAVB, propagating along the optical axis in a uniaxial crystal, can excite a right-hand circular polarized (RHCP) component superimposed with an on-axis vortex of topological charge (TC) number of 2. When the incident beam is an LHCP CAB imposed with an on-axis vortex of TC number of *l* = 1, both of the two components have an axisymmetric intensity distribution during propagation and form hollow beams near the focal plane because of the phase singularity. The phase pattern shows that the LHCP component carries an on-axis vortex of TC number of *l* = 1, while the RHCP component carries an on-axis vortex of TC number of *l* = 3. With a larger TC number (*l* = 3), the RHCP component has a larger hollow region in the focal plane compared to the LHCP component. We also studied cases of CABs imposed with one and two off-axis OVs. The off-axis OV makes the CAVB’s profile remain asymmetric throughout the propagation. As the propagation distance increases, the off-axis OVs move near the center of the beam and overlap, resulting in a special intensity and phase distribution near the focal plane.

## 1. Introduction

Circular airy beams (CABs), i.e., circular symmetric beams with an airy radial profile, were first proposed by Efremidis et al. in 2010 [1] and have received great attention due to their “abruptly autofocusing” property [1,2,3,4,5]. The “abruptly autofocusing” property enables the CAB to deliver high-energy pulses into transparent samples without damage before the focus [6], which is helpful in biomedical treatment [7], transparent material processing, and nonlinear optical processes [6]. The optical vortex (OV) was first proposed by Coullet P. in 1989, inspired by hydrodynamic vortices [8]. An OV beam has a phase singularity, a helical structure in the isophase plane, and carries orbital angular momentum (OAM) [9,10]. The nature of the OV beam carrying OAM makes multi-channel transmission possible, which increases the transmission capacity and communication rate of the system and also provides higher confidentiality [11]. The OV beam can transfer its own OAM to the particle, thus causing the particle to rotate and act as an “optical spanner” [12,13]. In addition, OV lasers can be used in material processing [14,15], such as in chiral metal nanoneedle formation [15].

Optical vortices (OVs) will translate, rotate, or annihilate in the background beam during the propagation due to the intensity gradient and phase gradient [16]. Dai H.T. et al. found that an airy beam with OVs will propagate along the parabolic trajectory with an acceleration velocity twice as fast as conventional airy beams before a critical position [17,18]. Since then, studies on Circular airy vortex beams (CAVBs) have emerged [19]. CAVBs possess the advantages of CABs and OV beams. Lu X.H. et al. found that OVs can greatly enhance the “abruptly autofocusing” property of a CAB, and the on-axis OV can cause the CAB to have a hollow intensity distribution [4]. These properties make CAVBs suitable for material processing and micromanipulation, such as laser inside-carving, laser hardening, and particle capture. On the other hand, it is an important to study the subject of propagation properties in optical beams in anisotropic media from both theoretical and applied points of view [20,21,22,23,24,25]. Ciattoni A. et al. found that a circularly polarized (CP) beam propagating along the optical axis in a uniaxial crystal can generate an OV with a topological charge (TC) number of 2 because of spin–orbital interactions [24,25]. The order of a Bessel beam propagating along the optical axes of a uniaxial crystal can be changed by two, which is meaningful for the generation of high-order Bessel beams possessing OVs [26,27]. It was found that only half of the energy is converted into a vortex beam at an infinite distance for a CP Gaussian beam [25,28]. Conversion efficiency can reach close to 100% on a small propagation distance along the axis of a uniaxial crystal for a Bessel beam [27]. In 2020, Ling X H et al. established a full-wave theory to describe beam propagation along the optical axis in a uniaxial crystal and revealed the physical origin of vortex generation [29]. Based on the plane-wave angular-spectrum theory, Ciattoni A. et al. developed an approach addressing paraxial propagation in a uniaxial crystal [24,25]. Inspired by the work of Ciattoni A., studies on the propagation of airy beams in uniaxial crystals have been reported [30,31,32,33]. These works mainly focused on cases that were orthogonal to the optical axis, in which the two intrinsic components of the beam are not coupled and no OV is generated. When a light beam propagates in a direction orthogonal to the optical axis, Cartesian field components parallel and orthogonal to the optical axis correspond to ordinary and extraordinary components, respectively, which are uncoupled [34]. The ordinary component exhibits standard Fresnel diffraction behavior, while the extraordinary component exhibits interesting anisotropic diffraction dynamics [30,31,32,33,34]. In 2017, we studied the propagation characteristics and the electro-optical coupling of a conventional CAB along the optical axis of a uniaxial crystal [35,36]. We found that the cylindrical symmetry of the CAB deteriorates during propagation in a uniaxial crystal, and the *n*_e_/*n*_o_ ratio has an important effect on its propagation characteristics [35]. Taking advantage of the electro-optical effect, one can enhance or weaken the “abruptly autofocusing” effect of a CAB [36]. To the best of our knowledge, there is no research that discusses the propagation of CABs with OVs along the optical axes of uniaxial crystals. In the following, we numerically investigate the propagation properties of CABs imposed different OVs in a uniaxial crystal.

## 2. Theory Model

The complex amplitude **E**(*r*) of the light field **E**(*r*) = Re[**E**(r)exp(-*iωt*)] propagating in an anisotropic medium obeys the following equation:

(1) $$\nabla^{2}E-\nabla (\nabla \cdot E)+k_{0}^{2}\varepsilon \cdot E=0$$

where *k_0_* = *ω*/*c* is the wave number in the vacuum and *ε* is the relative dielectric tensor. In the Cartesian coordinate system, *z*-axis is taken to be the optical axis of the uniaxial crystal. We assume that the light wave propagates along the optical axis, or the z-axis. The relative dielectric tensor of the uniaxial crystal is:

(2) $$\varepsilon =\left[\begin{array}{ccc} n_{o}^{2} & 0 & 0\\ 0 & n_{o}^{2} & 0\\ 0 & 0 & n_{e}^{2} \end{array}\right],$$

where *n*_o_ and *n*_e_ are the ordinary and extraordinary refractive indices of the uniaxial crystal, respectively. Based on the angular spectrum theory, Ciattoni A. proposed a method to deal with the problem of light propagation in uniaxial crystals [25]. The main result of this method is that the transverse component of an input light field at *z* = 0 gives rise to a light field inside the crystal that is a linear superposition of two parts, the ordinary and the extraordinary [25]:

(3) $$E(r,z)=\exp(ik_{0}n_{o}z)[A_{o}(r,z)+A_{e}(r,z)],$$

where **r** is the position vector at any transverse location, and $A_{o}(r,z)$ and $A_{e}(r,z)$ are given by:

$$A_{o}(r,z)=\int \frac{d^{2}k}{k^{2}}\left[\begin{array}{cc} k_{y}^{2} & -k_{x}k_{y}\\ -k_{x}k_{y} & k_{x}^{2} \end{array}\right]\tilde{E}(k)\cdot \exp(ik\cdot r-\frac{ik^{2}}{2k_{0}n_{o}}z),$$

(4) $$A_{e}(r,z)=\int \frac{d^{2}k}{k^{2}}\left[\begin{array}{cc} k_{x}^{2} & k_{x}k_{y}\\ k_{x}k_{y} & k_{y}^{2} \end{array}\right]\tilde{E}(k)\cdot \exp(ik\cdot r-\frac{in_{o}k^{2}}{2k_{0}n_{e}^{2}}z).$$

In Equations (4), $\tilde{E}(k)$ is the two-dimensional Fourier transform (FT) of the transverse field at *z* = 0, given by:

(5) $$\tilde{E}(k)=\frac{1}{{\left(2\pi \right)}^{2}}\int d^{2}r\exp(-ik\cdot r)E(r,0),$$

where **k** is the angular frequency vector. In order to obtain a more suitable solution representation for a CP incident field, two complex unit vectors, ${\hat{e}}_{+}=\sqrt{2}/2({\hat{e}}_{x}+i{\hat{e}}_{y})$ and ${\hat{e}}_{-}=\sqrt{2}/2({\hat{e}}_{x}-i{\hat{e}}_{y})$, corresponding to left- and right-hand CP light waves, are introduced. The left- and right-hand CP components of the whole light field in the crystal, $A_{+}(r,z)$ and $A_{-}(r,z)$, are given as [25]:

$$A_{+}(r,z)=F_{+}(r,z)+G_{-}(r,z),$$

(6) $$A_{-}(r,z)=F_{-}(r,z)-G_{+}(r,z),$$

where

(7) $$F_{\pm }(r,z)=\frac{1}{2}\int_{0}^{\infty }d^{2}k\exp(ik\cdot r)\left[\exp\left(-\frac{ik^{2}}{2k_{0}n_{o}}z\right)+\exp\left(-\frac{in_{0}k^{2}}{2k_{0}n_{e}^{2}}z\right)\right]{\tilde{E}}_{\pm }(k),$$

(8) $$G_{\pm }(r,z)=\frac{1}{2}\int_{0}^{\infty }d^{2}k\exp(ik\cdot r)\frac{k_{x}\pm ik_{y}}{k^{2}}\left[\exp\left(-\frac{ik^{2}}{2k_{0}n_{o}}z\right)-\exp\left(-\frac{in_{0}k^{2}}{2k_{0}n_{e}^{2}}z\right)\right]{\tilde{E}}_{\pm }(k).$$

In Equations (3)–(8), we can see that the transverse field distribution in any plane can be obtained as long as the FT of the incident light field, $\tilde{E}(k)$, is known.

## 3. Numerical Study

The complex amplitude, *E*(*r*), of an incident CAB imposed with OVs in a cylindrical coordinate can be expressed as [4,37]:

(9) $$E(r,\varphi ,z=0)=C\cdot Ai(\frac{r_{0}-r}{w})\exp(a\frac{r_{0}-r}{w}){\left(re^{i\varphi }-r_{k}e^{i\varphi_{k}}\right)}^{l},$$

where C is a constant, *Ai* represents the airy function, *r*_0_ is the initial radius of the CAB, *w* is the radial scale coefficient, *a* is the decay parameter, ($r_{k}$, $\varphi_{k}$) denotes the location of the OV, and *l* represents the TC number of the OV. Although the closed-form approximation of the FT of the CAB is given by relying on a suitable plane wave angular spectrum representation of the beam [5], there is no analytic expression for the FT of the CAB with OVs. Therefore, we employ the discrete Fourier transform to obtain the FT of the initial beam using a fast Fourier transform algorithm. In our numerical study, the beam parameters are given as follows: *r_0_* = 0.5 mm, *w* = 25µm, *a* = 0.1, and the wavelength λ = 632.8 nm, and the ordinary and extraordinary refractive indices of the uniaxial crystal are *n_o_* = 2.616 and *n*_e_ = 2.903, respectively.

### 3.1. CAB with On-Axis OV

First, we study the propagation characteristics of a left-hand circular polarized (LHCP) CAB imposed with an on-axis OV, and the TC, *l*, is 1. The incident light field can be expressed as:

(10) $$E(r,\varphi ,z=0)=C\cdot Ai(\frac{r_{0}-r}{w})\exp(a\frac{r_{0}-r}{w})re^{i\varphi }{\hat{e}}_{+}.$$

**Figure 1:**
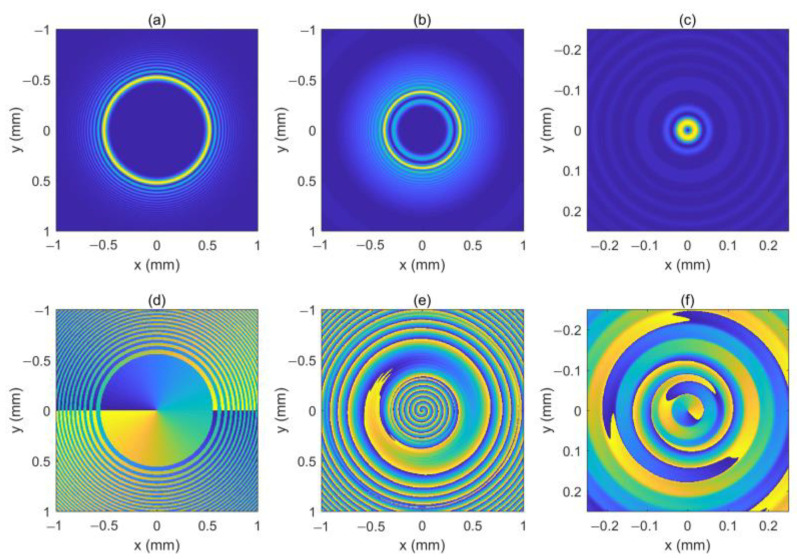
The intensity pattern and phase distribution of the left-hand circular polarized (LHCP) component at different distances. (**b**,**c**) show the intensity pattern for *z* = 100 mm and *z* = 200 mm, respectively; (**e**,**f**) show the phase distribution for *z* = 100 mm and *z* = 200 mm, respectively. The intensity pattern and phase distribution of the initial beam are also shown in (**a**,**d**).

**Figure 2:**
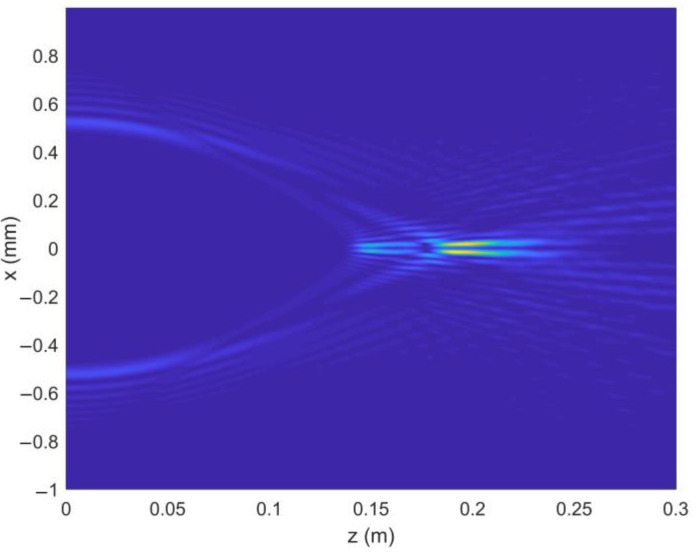
The propagation dynamics of the LHCP component.

**Figure 3:**
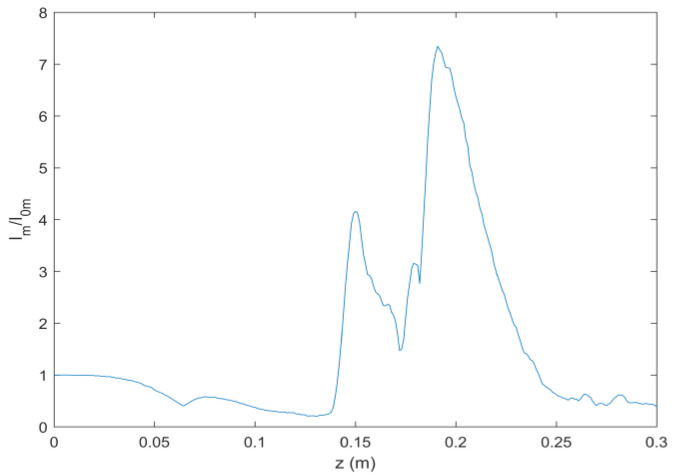
The “abruptly autofocusing” property of the LHCP component.

**Figure 4:**
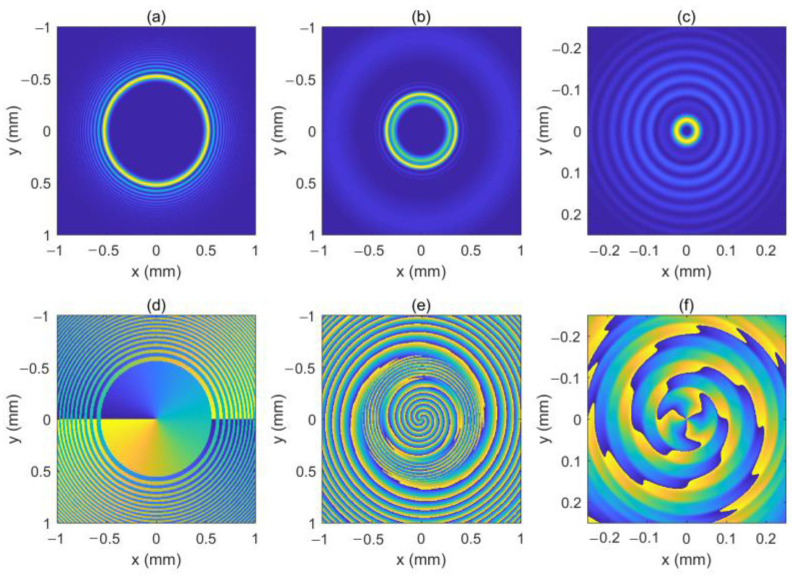
The intensity pattern and phase distribution of the right-hand circular polarized (RHCP) component at different distances. (**b**,**c**) show the intensity pattern for *z* = 100 mm and *z* = 180 mm, respectively; (**e**,**f**) show the phase distribution for *z* = 100 mm and *z* = 180 mm, respectively. The intensity pattern and phase distribution of the initial beam are also shown in (**a**,**d**).

**Figure 5:**
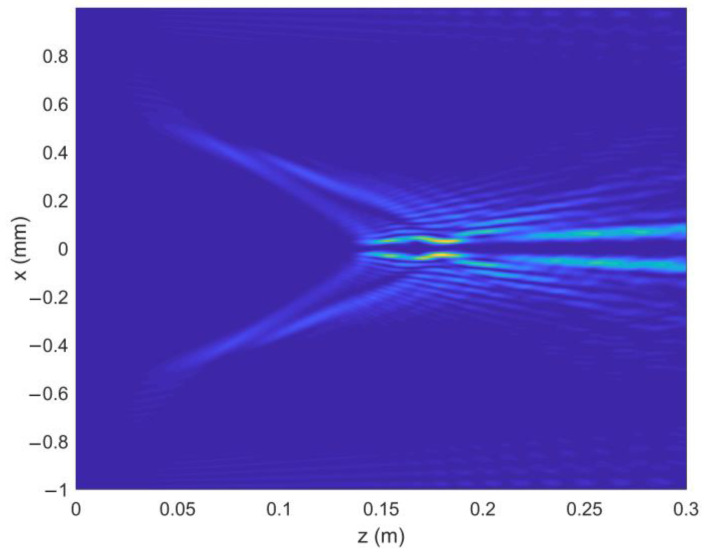
The propagation dynamics of the RHCP component.

**Figure 6:**
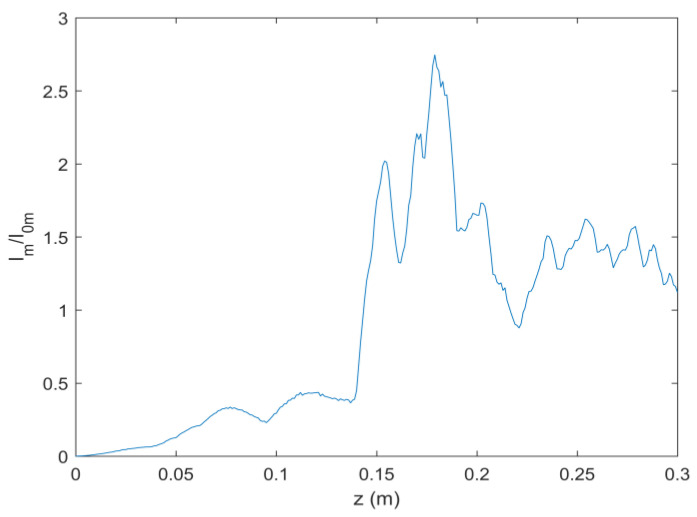
The “abruptly autofocusing” property of RHCP component.

As can be seen in Equation (6), the incident light field gives rise to LHCP and right-hand circular polarized (RHCP) components in the crystal. Figure 1 shows the intensity pattern and phase distribution of the LHCP component at a different distance (*z* = 0 mm, 100 mm and 200 mm). We can see from Figure 1a–c that the intensity distribution of the LHCP component remains axisymmetric, and the radius of the brightest ring gradually becomes smaller, showing an “abruptly autofocusing” property. Figure 1d–f shows that the LHCP component carries an on-axis vortex phase whose TC number, *l*, is 1 throughout the propagation. The evolution of the LHCP component in the uniaxial crystal is obtained as shown in Figure 2. Figure 2 shows that the LHCP component appears to have an “abruptly autofocusing” effect twice, which can be interpreted in Equation (7). In Equation (7), the item $\left[\exp\left(-\frac{ik^{2}}{2k_{0}n_{o}}z\right)+\exp\left(-\frac{in_{0}k^{2}}{2k_{0}n_{e}^{2}}z\right)\right]$ can be regarded as the propagation function, *H*, in angular theory. The propagation function, *H*, has two parts, causing the “abruptly autofocusing” effect to occur twice. Due to the vortex phase, the intensity profile of the LHCP component near the focal plane is hollow. The ratio *I*_m_/*I*_0m_ is introduced to study the “abruptly autofocusing” effect, where *I*_0m_ is the maximum intensity at the initial plane and *I*_m_ is the maximum intensity at an arbitrary transverse plane. The ratio *I*_m_/*I*_0m_ vs. propagation distance, *z*, is provided in Figure 3. Figure 3 also shows that the “abruptly autofocusing” effect appears twice, focal planes of which are at *z* = 150 mm and *z* = 192 mm. Because the portion of the energy from the incident light is coupled to the RHCP component, the ratio *I*_m_/*I*_0m_ is lower than 10, which is much lower than that of common CABs. Next, we studied the case of the RHCP component. Figure 4 provides the intensity pattern and phase distribution of the RHCP component at *z* = 100 mm and *z* = 180 mm. From Figure 4, one can see that the RHCP component also exhibits an “abruptly autofocusing” effect. Figure 4e,f shows that the RHCP component carries a vortex phase whose TC number is 3. This is because the RHCP component acquires a vortex phase with a TC number of 2 due to the spin reversal when the LHCP component is converted to an RHCP component [23,24]. Figure 5 shows the propagation dynamics of the RHCP component. As can be seen in Figure 5, near the focal plane, the beam is hollow, and the hollow region is greater than that of the LHCP component due to the larger TC number. The “abruptly autofocusing” property of the RHCP component is shown in Figure 6. From Figure 6, we can see that the maximum light intensity of RHCP appears at *z* = 180 m, which is different from that of the LHCP component.

### 3.2. CAB with Off-Axis OVs

First, we consider an LHCP CAB imposed with an off-axis OV, of which the TC, *l*, is 1. Therefore, the incident light field can be expressed as:

(11) $$E(r,\varphi ,z=0)=C\cdot Ai(\frac{r_{0}-r}{w})\exp(a\frac{r_{0}-r}{w})(re^{i\varphi }-r_{k}e^{i\varphi_{k}}){\hat{e}}_{+},$$

**Figure 7:**
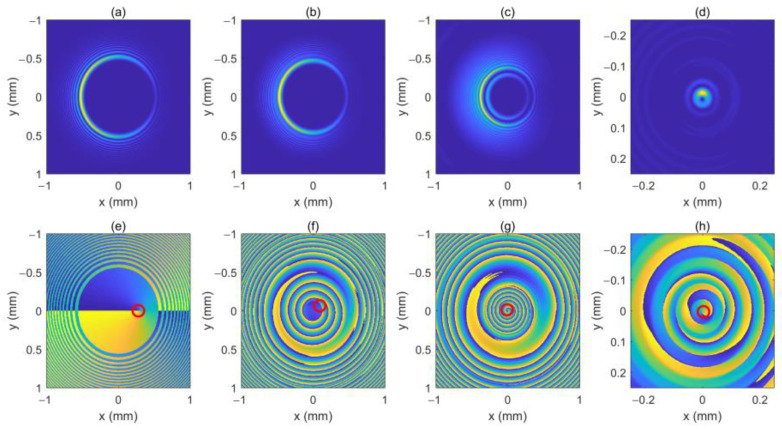
The intensity pattern and phase distribution of the LHCP component at different distances. (**b**–**d**) show the intensity pattern for *z* = 50 mm, *z* = 100 mm, and *z* = 200 mm, respectively; (**f**–**h**) show the phase distribution for *z* = 50 mm, *z* = 100 mm and *z* = 200 mm, respectively. The intensity pattern and phase distribution of the initial beam are also shown in (**a**,**e**). The center of the OV is marked with a red circle.

**Figure 8:**
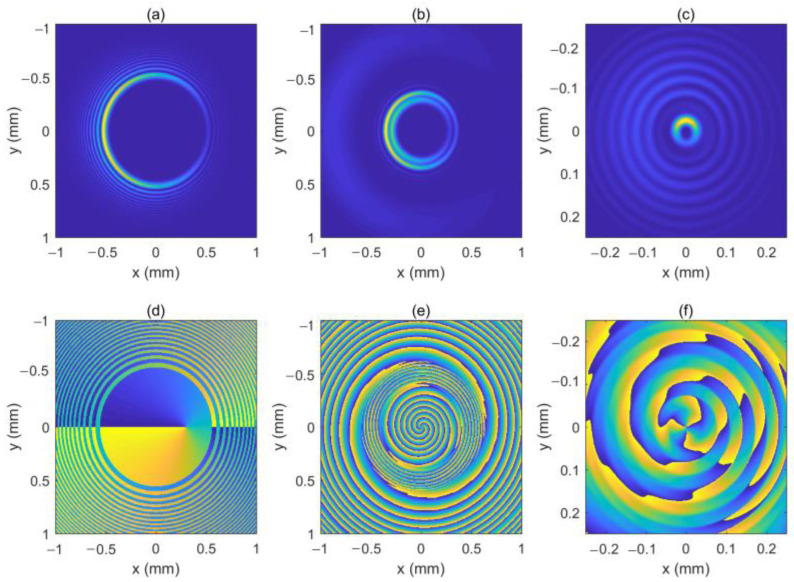
The intensity pattern and phase distribution of the RHCP component at different distances. (**b**,**c**) show the intensity pattern for *z* = 100 mm and *z* = 180 mm, respectively; (**e**,**f**) show the phase distribution for *z* = 100 mm and *z* = 180 mm, respectively. The intensity pattern and phase distribution of the initial beam are also shown in (**a**,**d**).

**Figure 9:**
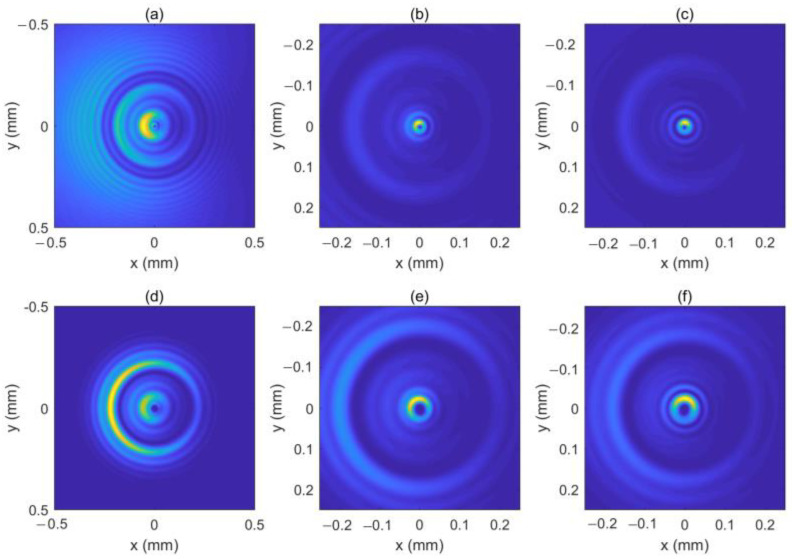
The intensity patterns of the LHCP component (**a**–**c**) and RHCP component (**d**–**f**) near the focal plane. (**a**,**c**), (**b**,**e**) and (**c**,**f**) show the intensity patterns for *z* = 140 mm, *z* = 145 mm, and *z* = 150 mm, respectively.

We assume the location of the OV is $r_{k}=0.3\;\mathrm{mm}$, $\varphi_{k}=0$. Figure 7 shows the intensity pattern and phase distribution of the LHCP component at different distances (*z* = 0 mm, 50 mm, 100 mm and 200 mm). From Figure 7a,e, we can see that the initial intensity pattern of the beam is not circularly symmetric and the OV is centered at *x* = 0.3 mm on the *x*-axis. In Figure 7e–h, one can see that the off-axis OV moves near the center of the beam because of the autofocusing property of the CAB, which is similar to the case of free space [18]. Figure 7c,g shows that the OV center has arrived at the center of the beam at *z* = 100 mm, while the “abruptly autofocusing” effect has not yet occurred. After the off-axis OV reaches the center of the beam, the beam seems to have acquired the phase of an on-axis OV with a TC number of 1, as shown in Figure 7g–h. Due to the off-axis OV, the intensity pattern of the LHCP component remains asymmetric throughout. Figure 8 shows the intensity pattern and phase distribution of the RHCP component at *z* = 100 mm and *z* = 180 mm, respectively. Since the RHCP component obtains an on-axis OV with a TC number of 2, it possesses a phase distribution similar to that of an on-axis OV with a TC number 3, as shown in Figure 8e,f. Interestingly, we found a rotation of the intensity pattern near the focal plane with the propagation distance. The intensity patterns of the LHCP and RHCP components near the focal plane are as shown in Figure 9. As can be seen in Figure 9, the light spot rotates from the left side to the upper side for both of the two components. This is because the intensity is concentrated on a small area due to the “abruptly autofocusing” effect, and the OV causes a redistribution of the intensity. This property has potential applications in particle manipulation and material processing.

Further, we study an LHCP CAB imposed with two off-axis OVs, both with a TC number of 1. The incident light field can be expressed as:

(12) $$E(r,\varphi ,z=0)=C\cdot Ai(\frac{r_{0}-r}{w})\exp(a\frac{r_{0}-r}{w})(re^{i\varphi }-r_{k1}e^{i\varphi_{k1}})(re^{i\varphi }-r_{k2}e^{i\varphi_{k2}}){\hat{e}}_{+}.$$

**Figure 10:**
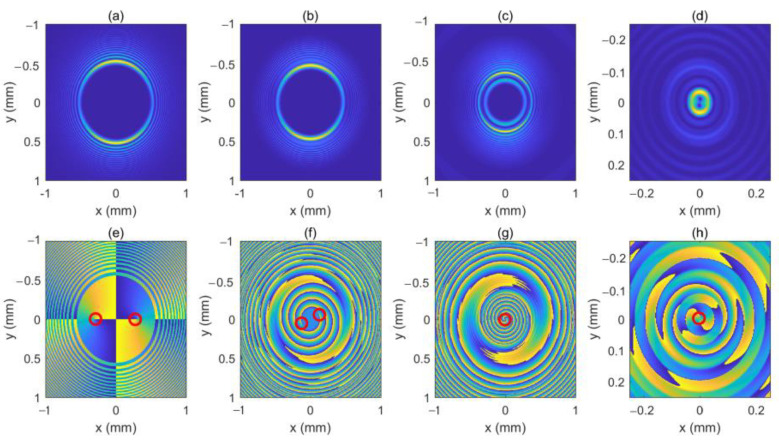
The intensity pattern and phase distribution of the LHCP component at different distances. (**b–d**) show the intensity pattern for *z* = 50 mm, *z* = 100 mm, and *z* = 200 mm; (**f**–**h**) show the phase distribution for *z* = 50 mm, *z* = 100 mm, and *z* = 200 mm. The intensity pattern and phase distribution of the initial beam are also shown in (**a**,**e**). The center of the optical vortex (OV) is marked with a red circle.

**Figure 11:**
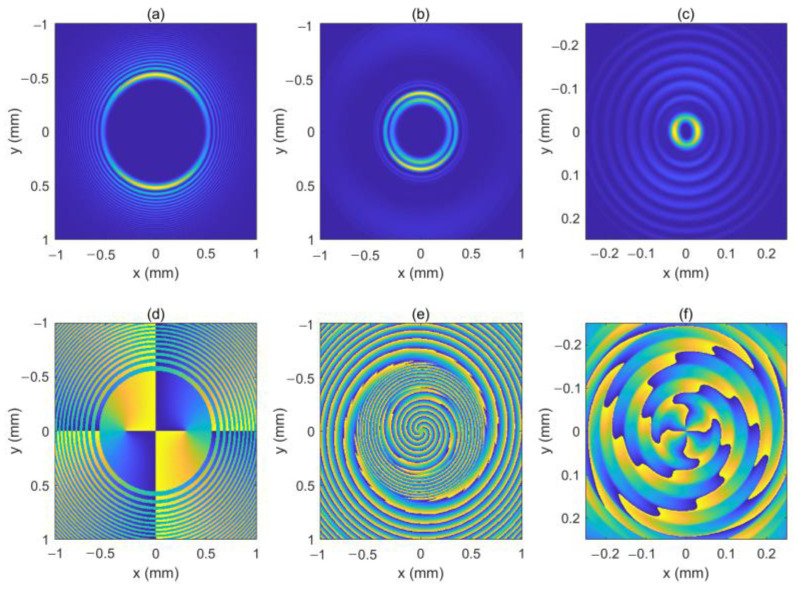
The intensity pattern and phase distribution of the RHCP component at different distances. (**b**,**c**) show the intensity pattern for *z* = 100 mm and *z* = 180 mm, respectively. (**e**,**f**) show the phase distribution for *z* = 100 mm and *z* = 180 mm, respectively. The intensity pattern and phase distribution of the initial beam are also shown in (**a**,**d**).

**Figure 12:**
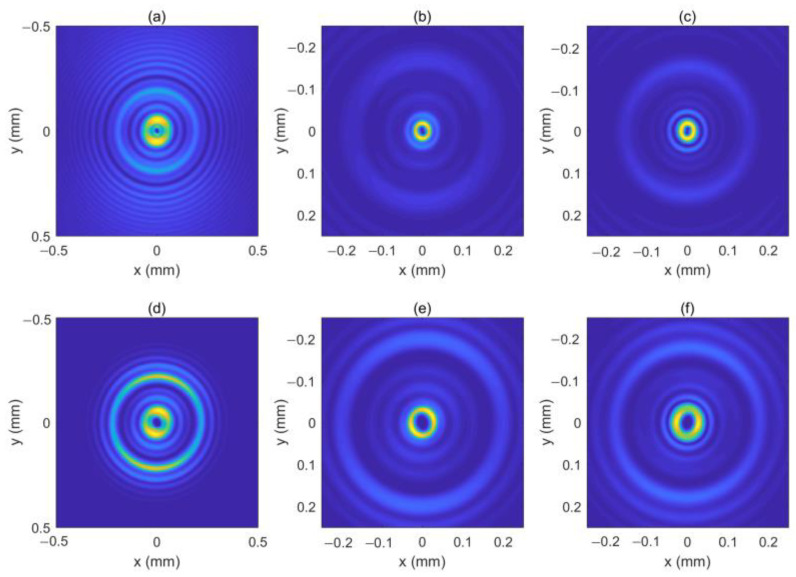
The intensity patterns of the LHCP component (**a**–**c**) and RHCP component (**d**–**f**) near the focal plane. (**a**,**c**), (**b**,**e**) and (**c**,**f**) show the intensity patterns for *z* = 140 mm, *z* = 145 mm, and *z* = 150 mm, respectively.

The locations of the two OVs are $r_{k1}=0.3\;\mathrm{mm},$ $\varphi_{k1}=0$ and $r_{k2}=-0.3\;\mathrm{mm}$, $\varphi_{k2}=0$, respectively. Figure 10 shows the intensity pattern and phase distribution of the LHCP component at *z* = 0 mm, *z* = 50 mm, *z* = 100 mm, and *z* = 200 mm, respectively. In Figure 10e–g, we can see that the two OVs move toward the center of the beam and superimpose on each other. Therefore, the LHCP component seems to have an on-axis OV with a TC number of 2, as shown in Figure 10g–h. Figure 11 shows the intensity pattern and phase distribution of the RHCP component at *z* = 100 mm, and *z* = 180 mm, respectively. Near the focal plane, the RHCP component seems to have an on-axis OV with a TC number of 4, as shown in Figure 11f. The two off-axis OVs make the intensity profiles of the two components asymmetric throughout. Further, we plot the intensity patterns of the LHCP and RHCP components near the focal plane, as shown in Figure 12. From Figure 12, we can see that the light spot undergoes rotation near the focal plane, which is similar to the case of one of the off-axis OVs.

## 4. Conclusions

In summary, we numerically investigated the propagation characteristics of an LHCP CAB imposed with different OVs along the optical axis of a uniaxial crystal. An LHCP CAB imposed with an on-axis OV will give rise to LHCP and RHCP components in the crystal, which have circularly symmetric distribution during propagation and form hollow beams near the focal plane. We also studied the effect of one and two off-axis OVs on the propagation characteristics of the CAB and obtained the intensity and phase distribution of the beam near the focal plane. Our results are instructive for expanding the application of CABs imposed with OVs.
